# Efficacy and safety of robotic versus laparoscopic liver resection for hepatocellular carcinoma: a propensity score-matched retrospective cohort study

**DOI:** 10.1007/s12072-024-10658-6

**Published:** 2024-05-13

**Authors:** He Li, Lingzhan Meng, Simiao Yu, Haocheng Zheng, Lingxiang Yu, Hongbo Wang, Hui Ren, Hu Li, Xiaofeng Zhang, Zizheng Wang, Peng Yu, Xiongwei Hu, Muyi Yang, Jin Yan, Yanling Shao, Li Cao, Xia Ding, Zhixian Hong, Zhenyu Zhu

**Affiliations:** 1 Department of Hepatobiliary Surgery, The Fifth Medical Center of PLA General Hospital, Beijing, 100039 China; 2 Dongzhimen Hospital, Beijing University of Chinese Medicine, Beijing, 100700 China; 3 Department of Hepatology of Traditional Chinese Medicine, The Fifth Medical Center of PLA General Hospital, Beijing, 100039 China

**Keywords:** Hepatocellular carcinoma (HCC), Robotic liver resection (RLR), Laparoscopic liver resection (LLR), Propensity score matching (PSM), Recurrence

## Abstract

**Background:**

Evidence concerning long-term outcome of robotic liver resection (RLR) and laparoscopic liver resection (LLR) for hepatocellular carcinoma (HCC) patients is scarce.

**Methods:**

This study enrolled all patients who underwent RLR and LLR for resectable HCC between July 2016 and July 2021. Propensity score matching (PSM) was employed to create a 1:3 match between the RLR and LLR groups. A comprehensive collection and analysis of patient data regarding efficacy and safety have been conducted, along with the evaluation of the learning curve for RLR.

**Results:**

Following PSM, a total of 341 patients were included, with 97 in the RLR group and 244 in the LLR group. RLR group demonstrated a significantly longer operative time (median [IQR], 210 [152.0–298.0] min vs. 183.5 [132.3–263.5] min; *p* = 0.04), with no significant differences in other perioperative and short-term postoperative outcomes. Overall survival (OS) was similar between the two groups (*p* = 0.43), but RLR group exhibited improved recurrence-free survival (RFS) (median of 65 months vs. 56 months, *p* = 0.006). The estimated 5-year OS for RLR and LLR were 74.8% (95% CI: 65.4–85.6%) and 80.7% (95% CI: 74.0–88.1%), respectively. The estimated 5-year RFS for RLR and LLR were 58.6% (95% CI: 48.6–70.6%) and 38.3% (95% CI: 26.4–55.9%), respectively. In the multivariate Cox regression analysis, RLR (HR: 0.586, 95% CI (0.393–0.874), *p* = 0.008) emerged as an independent predictor of reducing recurrence rates and enhanced RFS. The operative learning curve indicates that approximately after the 11th case, the learning curve of RLR stabilized and entered a proficient phase.

**Conclusions:**

OS was comparable between RLR and LLR, and while RFS was improved in the RLR group. RLR demonstrates oncological effectiveness and safety for resectable HCC.

**Supplementary Information:**

The online version contains supplementary material available at 10.1007/s12072-024-10658-6.

## Introduction

Primary liver cancer (PLC) is the sixth most common cancer worldwide and the third most common cause of cancer death, with hepatocellular carcinoma (HCC) accounting for about 85% of all PLC [1]. Despite the rapid advancements in medical technology, radical surgical resection of the primary tumor remains the crucial component in the treatment of HCC. Robotic liver resection (RLR) is a relatively novel technique and integrates the benefits of traditional surgery with the precision and flexibility of robotic surgical systems, the question of whether RLR outperforms traditional surgery in clinical practice remains ongoing.

Recent high-quality retrospective and prospective studies have corroborated the safety and efficacy of RLR in both short- and long-term outcomes, demonstrating its superiority over open liver resection (OLR) for liver cancer patients [2, 3]. Regarding the comparison of RLR and laparoscopic liver resection (LLR), the existing high-quality retrospective studies predominantly focus on short-term prognosis, with a severe lack of long-term outcomes concerning tumors [4, 5].

Our ultimate aim was to evaluate the potential impact of RLR on the long-term outcome of HCC patients in a single high-volume center in China, and concurrently evaluated the learning curve of this technique for HCC treatment. We present the following article in accordance with the STROBE reporting checklist.

## Patients and methods

### Patients

This cohort study entailed a retrospective analysis of databases meticulously maintained by the largest specialized liver disease medical center in China, The Fifth Medical Center, General Hospital of PLA. The study focused on patients who underwent RLR for HCC (RLR group) between July 1, 2016 and July 1, 2021. This cohort was compared with a control group of patients who underwent LLR for HCC (LLR group) during the same time frame. Prior to surgery, all patients provided informed consent, authorizing the anonymized collection of data and audiovisual recording of the surgical procedure. This retrospective cohort study was received approval from the institutional review board and ethics committee of The Fifth Medical Center, General Hospital of PLA (protocol KY-2022-12-76-1). This study was registered was registered as a retrospective cohort study at Research Registry (UIN: researchregistry9622, available at https://www.researchregistry.com) in compliance with the World Medical Association’s Declaration of Helsinki, 2013.

### Definitions

The diagnosis of HCC was established using computed tomography or magnetic resonance imaging, in accordance with internationally approved radiological standards [6, 7]. The study excluded cases meeting the following criteria: (1) heterogeneous invasive lesions upon final pathology (e.g., adenosquamous carcinoma, etc.); (2) secondary carcinoma of the liver; (3) history of previous liver resection; (4) patients who required concomitant procedures, such as lymph-node dissection around the porta hepatis, cryoablation, radiofrequency ablation, or biliary tract exploration were not included in this study.

All robotic surgeries were performed using the da Vinci Si Surgical System (Intuitive Surgical, Sunnyvale, CA, USA). The surgical approaches of RLR and LLR were conducted using the procedures as previously described [8, 9].

The baseline characteristics examined in both study groups encompassed age, sex, body mass index (BMI), Child–Pugh–Turcotte (CPT) status, age-adjusted Charlson Comorbidity Index (aCCI) [10], American Society of Anesthesiologists (ASA) physical status score [11], IWATE criteria difficulty [12], Eastern Cooperative Oncology Group performance status (ECOG PS) score, albumin–bilirubin (ALBI) score [13], hemoglobin (HGB), platelet count (PLT), carbohydrate antigen 19-9 (CA19-9), carbohydrate antigen 125 (CA125), carcinoembryonic antigen (CEA), and alpha fetoprotein (AFP). The relationship between surgical approach and study outcomes, such as OS and RFS, was investigated using directed acyclic graphs (DAGs) to identify potential confounders (Appendix Fig. 3).

Perioperative outcomes comprised estimated blood loss (EBL), requirement for packed red blood cell (pRBC) transfusion, conversion rate to open laparotomy, operative time, duration of stay in the intensive care unit (ICU), and the length of postoperative hospital stay.

Short-term postoperative outcomes were meticulously documented, encompassing common complications (such as hepatic failure, fever, abdominal effusion, pleural effusion, and abdominal infection), along with 30-day morbidity, 30-day reoperation rate, and 90-day mortality were recorded in detail. Moreover, the severity of complications was classified using the Clavien–Dindo classification (CDC) [14].

We adhered to a standardized surveillance protocol, conducting postoperative follow-up at 30 days after the operation and subsequent patient assessments at 3-month intervals. Tumor markers, including CA 19-9, CA 125, and carcinoembryonic antigen (CEA), were assessed every 3 months, alongside a chest–abdomen–pelvis computed tomography (CT-CAP) performed at the same intervals. Comprehensive details pertaining to tumor recurrence and progression were collected using both the institutional database and patient information obtained from collaborating local hospitals.

The long-term oncological outcomes, including patients’ overall survival (OS) and recurrence-free survival (RFS), were the primary endpoints of this study. OS was defined as the duration from diagnosis to patient death or the last date of follow-up (cut-off date: August 1, 2023), while RFS was defined as the period from surgical intervention to the known date of disease recurrence or the last follow-up date (cut-off date: August 1, 2023).

### Statistical analysis

Continuous data that follow a normal distribution are reported in terms of the mean (SD), while continuous data that do not follow a normal distribution are presented using the median (IQR). Categorical data are reported as counts and percentages. Comparisons between the OLR and RLR groups were performed using the Student’s *t* test or Mann–Whitney *U* test for continuous variables, Chi-squared test or Fisher exact test for categorical variables, and Cochran–Armitage test for trend for ordinal variables. Balance in covariates between treatment groups was also evaluated by the standardized mean difference (SMD). An SMD of less than 0.1 was deemed to be the ideal balance.

Randomization is sometimes difficult to achieve in observational studies. In this study, the cost of robotic surgery exceeded that of laparoscopic surgery, falling outside the coverage of basic medical insurance. Consequently, there was potential bias in the patient’s choice of surgical approach. Moreover, non-randomized grouping caused an imbalance between groups in terms of baseline features. In this context, propensity score matching (PSM) effectively reduces the confounding bias and obtains effects similar to those of randomized controlled studies. To achieve the balance between groups while making full use of the collected data, we employed a one-to-three nearest-neighbor matching algorithm with a caliper of 0.1. During the matching process, R software selects three patients with the closest propensity scores in the LLR group to match with one patient in the RLR group. However, due to the smaller size of the RLR group, only some patients in this group could find all three matched patients, resulting in an imperfect 1:3 ratio. Although the proportions are not strictly accurate, the matching model remains valid provided that it is ensured that the two matched groups are as consistent as possible on critical confounding variables, thereby conferring high internal validity to the results. Standardized mean differences (SMD) were estimated before and after matching to evaluate the balance of covariates, and the value of SMD less than 0.1 was considered relatively balanced enough. The propensity score was estimated using a multivariable logistic regression, with type of surgery as the dependent variable and age, sex, BMI, CPT status, aCCI, ASA physical status score, IWATE criteria difficulty, ECOG PS score, ALBI score, HGB, platelet count, CA19-9, CA125, CEA, and AFP as covariates.

Survival analysis of OS and RFS was performed by log-rank test and plotted by the Kaplan–Meier curve. Factors found to be significant in the univariate analysis and potential confounders were entered into the multivariate Cox regression analysis. Hazard ratio (HR) and its confidence interval (CI) were also calculated using Cox proportional hazard analysis. The PH assumptions of Cox proportional risk regression analysis were evaluated by Schoenfeld residual method.

CUSUM analysis is a statistical technique applied to surgical procedures for the quantitative estimation of the learning curve [15, 16]. The standard CUSUM analysis shows the cumulative differences between the observed data and the target value. To perform multidimensional CUSUM analysis, we designated operative time, estimated blood loss, postoperative complication, and postoperative hospital length of stay as the assessment indicators of surgical competence. These four assessment indicators were, respectively, set as quantized value $\delta _1$, $\delta _2$, $\delta _3$, and $\delta _4$ for each case. The quantized value of assessment indicator was defined as $\delta$ = $X_n - X_0$, where $X_n$ was an individual attempt following the nth procedure, with $X_n$ = 1 if a failure occurred and $X_n$ = 0, if it did not. $X_0$ is the established risk or failure rate of the control to which the ongoing attempts are compared. $X_0$ can be calculated either as an overall frequency if this is known in this case, or on a case-by-case basis as with paired control trials. In this study, the mean or median of RLR group data after PSM is taken as the target value, and the proportion of each index reaching the target value is calculated to obtain the overall frequency. The $X_0$ for these four assessment indicators were, respectively, 0.390, 0.537, 0.439, and 0.512. Therefore, the quantized value of surgical competence for each case was defined as *S*=$\delta _1$+$\delta _2$+$\delta _3$+$\delta _4$. After each case, scores were sequentially added and then plotted graphically by the equation: ${\text {CUSUM}} = \sum$ $S_i$. It was based on CUSUM graph that fit a restricted cubic spline (RCS) curve was used to depict the learning curve. A positive slope signified that the desired target remained unattained, while a negative slope indicated that it had been surpassed. The pivot point where the slope transitioned from positive to negative served as a reflection of the surgical procedure’s proficiency.

A 2-sided *p* < 0.05 was considered significant in all the analysis. All statistical analyses were performed using R software (version.4.3.1).

## Results

### Clinical characteristics of patients

Over a 5-year period, a total of 529 patients were included in the analysis, consisting of 107 individuals in the RLR group (85 men, 22 women) and 422 in the OLR group (348 men, 74 women). The baseline demographic and clinical characteristics for this cohort are detailed in Table 1. In the initial analysis, it was observed that patients who underwent RLR exhibited a significantly lower ASA score (*p* = 0.03). Additionally, there was a notable difference in the IWATE criteria difficulty between the RLR and OLR groups prior to PSM (*p* = 0.04). Patients in the RLR group also presented with a significantly lower ECOG PS score compared to the LLR group (*p* = 0.01). However, the CPT status of the RLR group was significantly higher than that of the LLR group (*p* = 0.01).

**Table 1:** Demographic and clinical characteristics of the study population before and after PSM

Variable	Before PSM				After PSM			
	RLR (*n* = 107)	LLR (*n* = 422)	*p*	SMD	RLR (*n* = 97)	LLR (*n* = 244)	*p*	SMD
Age, mean (SD), years	52.54 (10.74)	54.28 (9.70)	0.13	0.161	53.02 (10.79)	53.73 (10.09)	0.43	0.047
Sex, no. (%)			0.46	0.074			0.60	< 0.001
Male	85 (79.4)	348 (82.5)			78 (80.4)	202 (82.8)		
Female	22 (20.6)	74 (17.5)			19 (19.6)	42 (17.2)		
BMI, median (IQR)	25.14 (22.79–26.70)	24.24 (22.38–26.88)	0.24	0.133	25.03 (22.49–26.78)	24.32 (22.52–26.89)	0.64	0.033
CPT status			0.01	0.198			0.50	0.022
0	96 (89.7)	404 (95.7)			90 (92.8)	231 (94.7)		
1	11 (10.3)	18 (4.3)			7 (7.2)	13 (5.3)		
aCCI, median (IQR)	5 (3–6)	5 (4–6)	0.44	0.089	5 (3–6)	5 (3–6)	0.57	0.015
ASA score			0.03	0.250			0.74	0.030
2	77 (72.0)	256 (60.7)			67 (69.1)	164 (67.2)		
3	30 (28.0)	166 (39.3)			30 (30.9)	80 (32.8)		
IWATE criteria difficulty			0.004	0.386			0.62	0.089
Low	16 (15.0)	117 (27.7)			16 (16.5)	46 (18.9)		
Intermediate	59 (55.1)	233 (55.2)			57 (58.8)	143 (58.6)		
$\ge$ Advanced	30 (28.0)	69 (16.4)			24 (24.7)	55 (22.5)		
ECOG PS score			0.001	0.606			0.55	0.016
0	102 (95.3)	353 (83.6)			92 (94.8)	228 (93.4)		
1	5 (4.7)	64 (15.2)			5 (5.2)	16 (6.6)		
$\ge$ 2	0 (0.0)	5 (1.2)			0	0		
ALBI score			0.19	0.168			0.40	0.064
1	37 (34.6)	119 (28.2)			33 (34.0)	76 (31.1)		
$\ge$ 2	70 (65.4)	303 (71.8)			64 (66.0)	168 (68.8)		
HGB, median (IQR), g/L	146 (134.5–158.0)	144.5 (135.0–155.0)	0.56	0.096	146.0 (135.0–158.0)	145.0 (135.75–154.25)	0.84	0.004
PLT, median (IQR), 10^9^/L	159.0 (119.0–192.50)	150.0 (120.25–184.0)	0.31	0.086	160 (122–192)	152.5 (123.75–192)	0.69	0.013
CA19–9, median (IQR), U/mL	15.67 (10.07–23.39)	12.43 (7.86–20.81)	0.62	0.063	14.38 (10.15–18.47)	11.60 (7.21–20.14)	0.33	0.047
CA125, median (IQR), U/mL	9.71 (7.68–12.62)	9.65 (6.86–14.19)	0.71	0.060	9.94 (7.56–12.72)	9.94 (6.93–14.19)	0.95	0.015
CEA, median (IQR), ng/mL	2.13 (1.43–3.36)	2.22 (1.49–3.24)	0.72	0.097	2.14 (1.59–3.50)	2.155 (1.48–3.21)	0.55	0.001
AFP, median (IQR), ng/mL	6.08 (3.93–243.90)	12.07 (3.69–212.23)	0.40	0.036	14.12 (3.73–274.0)	13.11 (3.63–234.63)	0.70	0.081

Data are presented as mean (SD), *n* (%) or median (IQR)

PSM, propensity score matching; BMI, body mass index; CPT, Child–Pugh–Turcotte; aCCI, age-adjusted Charlson Comorbidity Index; ASA, American Society of Anesthesiologists; ECOG PS, Eastern Cooperative Oncology Group performance status; ALBI, albumin bilirubin; HGB, hemoglobin; PLT, platelet count; CA19-9, carbohydrate antigen 19-9; CA125, carbohydrate antigen 125; CEA, carcinoembryonic antigen; AFP, alpha fetoprotein

PSM is a statistical method employed to address causal inference problems. It achieves this by seeking observations from the experimental and control groups with similar propensity scores, thereby reducing the impact of potential confounding variables on causal estimation. After PSM involving 341 patients across all groups, no significant differences were observed between the two groups in terms of both baseline and pathologic characteristics. The SMD for all matched covariates was < 0.1 after PSM, signifying that weighted patient cohorts in both groups were comparable.

Regarding the oncological characteristics of the resected specimen, no significant differences were found between the RLR and LLR groups (Table2).

**Table 2:** Clinicopathological characteristics oncologic features before and after PSM

Variable	Before PSM			After PSM		
	RLR (*n* = 107)	LLR (*n* = 422)	*p*	RLR (*n* = 97)	LLR (*n* = 244)	*p*
Cirrhosis			0.75			0.87
No	17 (15.9)	75 (17.8)		16 (16.5)	42 (17.2)	
Yes	90 (84.1)	347 (82.2)		81 (83.5)	202 (82.8)	
Etiology			0.85			0.68
HBV	94 (87.9)	373 (88.4)		84 (86.6)	220 (90.2)	
HCV	5 (4.7)	18 (4.3)		5 (5.2)	9 (3.7)	
Alcohol	3 (2.8)	17 (4.0)		3 (3.1)	8 (3.3)	
Others	5 (4.7)	14 (3.3)		5 (5.2)	7 (2.9)	
Tumor size, median (IQR), cm	3.5 (2.0–4.7)	3.0 (2.0–4.5)	0.36	3.5 (2–4.7)	3.05 (2–5)	0.91
Tumor number			0.28			0.36
1	99 (92.5)	402 (95.3)		89 (91.8)	229 (93.9)	
2	6 (5.6)	15 (3.6)		6 (6.2)	13 (5.3)	
$\ge$ 3	2 (1.9)	5 (1.1)		2 (2.1)	2 (0.8)	
Grade			0.37			0.21
G1	2 (1.9)	20 (4.7)		2 (2.1)	16 (6.6)	
G2	102 (95.3)	389 (92.2)		93 (95.9)	222 (91.0)	
G3	3 (2.8)	13 (3.1)		2 (2.1)	6 (2.5)	
Surgical margin, no. ($\%$)			0.18			0.14
Negative	105 (98.1)	402 (95.3)		96 (99.0)	234 (95.9)	
Positive	2 (1.9)	20 (4.7)		1 (1.0)	10 (4.1)	
Surgical margin distance, median (IQR), cm	0.5 (0.1–1)	0.5 (0.13–1.5)	0.07	0.5 (0.1–1)	0.6 (0.18–1.5)	0.06
Microvascular invasion			0.17			0.14
−	9 (8.4)	92 (21.8)		8 (8.2)	61 (25.0)	
+	86 (80.4)	262 (62.1)		78 (80.4)	140 (57.4)	
++	12 (11.2)	68 (16.1)		11 (11.3)	43 (17.6)	
CD34			0.74			0.58
−	4 (3.7)	14 (3.3)		4 (4.1)	8 (3.3)	
+	103 (96.3)	407 (96.4)		93 (95.9)	235 (96.3)	
++	0 (0.0)	1 (0.2)		0 (0.0)	1 (0.4)	
GPC-3, median (IQR)			0.37			0.12
−	7 (6.5)	43 (10.2)		7 (7.2)	34 (13.9)	
+	93 (86.9)	352 (83.4)		84 (86.6)	197 (80.7)	
++	7 (6.5)	27 (6.4)		6 (6.2)	13 (5.3)	

GPC-3, glypican-3

### Perioperative outcomes and short-term postoperative outcomes

The perioperative outcomes for both groups are detailed in Table 3. After PSM, the RLR group showed a higher operative time (median [IQR], 210 [152.0–298.0] mins vs 183.5 [132.3–263.5] mins, *p* = 0.04) compared to the LLR group.

**Table 3:** Perioperative outcomes and short-term postoperative outcomes before and after PSM

Variable	Before PSM			After PSM		
	RLR (*n* = 107)	LLR (*n* = 422)	*p*	RLR (*n* = 97)	LLR (*n* = 244)	*p*
EBL, median (IQR), mL	100 (50–300)	100 (50–300)	0.15	100 (50–300)	100 (50–300)	0.35
No. of pRBC units			0.21			0.52
0	99 (92.5)	403 (95.5)		91 (93.8)	233 (95.5)	
$\ge$ 1	8 (7.5)	19 (4.5)		6 (6.2)	11 (4.5)	
Operative time, median (IQR), mins	212.0 (153.0–299.5)	179.0 (126.25–248.0)	< 0.001	210.0 (152.0–298.0)	183.50(132.25–263.50)	0.04
Conversion to laparotomy			0.15			0.05
No	104 (97.2)	395 (93.6)		95 (97.9)	226 (92.6)	
Yes	3 (2.8)	27 (6.4)		2 (2.1)	18 (7.4)	
CDC			0.23			0.28
1	103 (96.3)	387 (91.7)		93 (95.9)	223 (91.4)	
$\ge$ 2	4 (3.7)	35 (8.3)		4 (4.1)	21 (8.6)	
Fever			< 0.001			< 0.001
< 37.3 $^{\circ }\hbox {C}$	80 (74.8)	394 (93.4)		73 (75.3)	230 (94.3)	
37.3–38.0 $^{\circ }\hbox {C}$	21 (19.6)	17 (4.0)		19 (19.6)	10 (4.1)	
38.1–39.0 $^{\circ }\hbox {C}$	5 (4.7)	10 (2.4)		4 (4.1)	4 (1.6)	
$\ge$ 39.1$^{\circ }\hbox {C}$	1 (0.9)	1 (0.2)		1 (1.0)	0 (0.0)	
Ascites			0.13			0.35
No	80 (74.8)	322 (76.3)		77 (79.4)	182 (74.6)	
Yes	27 (25.2)	100 (23.7)		20 (20.6)	62 (25.4)	
Pleural effusion			0.43			0.35
No	76 (71.0)	283 (67.1)		69 (71.1)	161 (66.0)	
Yes	31 (29.0)	139 (32.9)		28 (28.9)	83 (34.0)	
Intra-abdominal infection			0.12			0.15
No	103 (96.3)	388 (91.9)		93 (95.9)	223 (91.4)	
Yes	4 (3.7)	34 (8.1)		4 (4.1)	21 (8.6)	
ICU length of stay			0.98			0.91
0	104 (97.2)	410 (97.2)		94 (96.9)	237 (97.1)	
$\ge$ 1	3 (2.8)	12 (2.8)		3 (3.1)	7 (2.9)	
Postoperative hospital length of stay, median (IQR), days	8 (7–9.5)	8 (7–10)	0.29	8 (7–9)	8 (7–10)	0.18
30-day readmission rate			0.41			0.72
No	95 (88.8)	362 (85.8)		86 (88.7)	213 (87.3)	
Yes	12 (11.2)	60 (14.2)		11 (11.3)	31 (12.7)	
30-day reoperation rate			0.7			0.67
No	95 (88.8)	380 (90.0)		86 (88.7)	220 (90.2)	
Yes	12 (11.2)	42 (10.0)		11 (11.3)	24 (9.8)	
90-day mortality			0.38			–
No	107 (100.0)	419 (99.3)		97 (100.0)	244 (100.0)	
Yes	0	3 (0.7)		0	0	
Cost of surgery, median (IQR), CNY	43,173.2 (26,236.8–58,170.15)	23,729.40 (16,289.21–31650.47)	< 0.001	42,465.60 (26,217.0–58,089.78)	25,297.35 (17,228.95–33,579.07)	< 0.001
Cost of anesthesia, median (IQR), CNY	6024.7 (5238.45–6965.35)	5492.70 (4686.03–6466.83)	0.001	6024.70 (5283.50–6915.70)	5631.35 (4764.62–6590.38)	0.02

EBL, estimated blood loss; pRBC, packed red blood cells.

In the postoperative phase, the RLR group exhibited a higher incidence of fever compared to the LLR group (*p* < 0.001). No significant differences were observed in other complications between the two groups. Patients who underwent RLR showed no significant differences in the length of postoperative hospital stay compared to those who underwent LLR (median [IQR], 8 [7–9] vs 8 [7–10] day; *p* = 0.18). Furthermore, the overall rates of 30-day readmission, 30-day reoperation, and 90-day mortality were similar between the groups.

Additionally, the health economics findings reveal that the surgical costs in the RLR group were significantly higher compared to the LLR group (*p* < 0.001). The cost of anesthesia was also notably elevated in the RLR group in contrast to the LLR group (*p* = 0.02).

To explore the efficacy of the RLR group and the LLR group in complex hepatectomy, we compared the primary perioperative and short-term outcomes associated with liver segments I, VII, and VIII. A combined analysis of these three segments was also performed (detailed in Appendix Tables 5, 6, 7, 8). Our statistical results showed that the length of postoperative hospital stays for segment I resection was significantly shorter in the RLR group than in the LLR group (*p* = 0.07). No significant differences were observed in other perioperative and short-term outcomes.

### Long-term oncologic outcomes

**Fig. 1:**
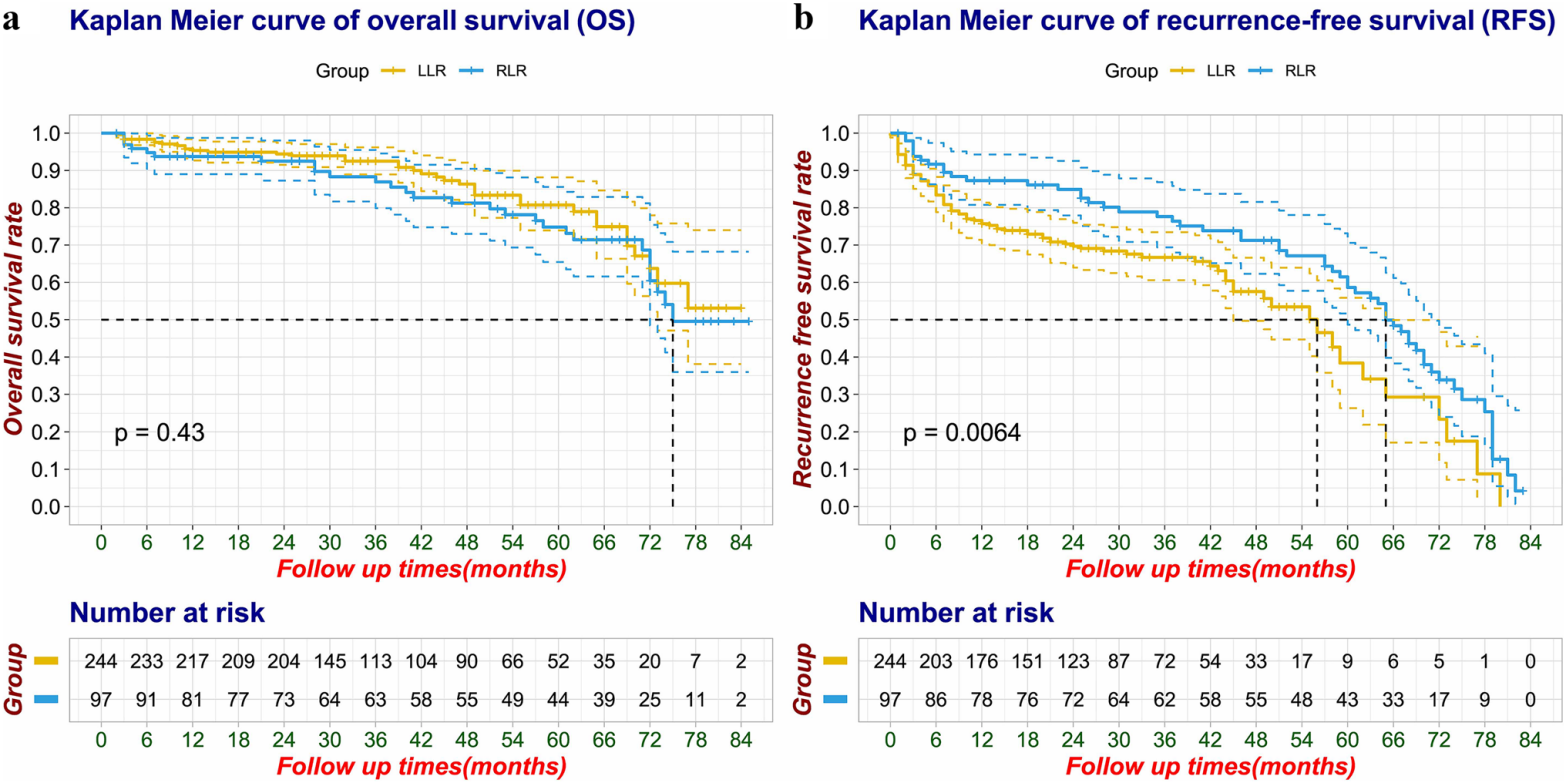
**A** Kaplan–Meier curve of overall survival (OS) after PSM (log-rank test, *p* = 0.43); **B** Kaplan–Meier curve of recurrence-free survival (RFS) after PSM (log-rank test, *p* = 0.006)

At a median follow-up duration of 49 months (IQR, 28–67 months), the median OS and RFS times were 76 and 57 months, respectively, for the entire cohort. Following PSM, no significant difference in OS was observed between the RLR and LLR groups among patients with HCC (*p* = 0.43). However, patients in the RLR group exhibited a longer median RFS duration compared to the LLR group (median of 65 months vs. 56 months, *p* = 0.006) (Fig. 1). The estimated 5-year OS for RLR and LLR were 74.8% (95% CI: 65.4–85.6%) and 80.7% (95% CI: 74.0–88.1%), respectively. The estimated 5-year RFS for RLR and LLR were 58.6% (95% CI: 48.6–70.6%) and 38.3% (95% CI: 26.4–55.9%), respectively.

Given the observed significant difference in RFS between patients undergoing RLR and those in the LLR group, a Cox proportional hazard analysis was conducted on the entire cohort. This aimed to further affirm the impact of the surgical approach on RFS and explore significant covariates. In the multivariate Cox regression analysis for recurrence outcome, RLR (HR: 0.586, 95% CI (0.393–0.874), *p* = 0.008) emerged as an independent predictor associated with decreased recurrence rates and improved RFS (Table 4). Additionally, an ECOG score $\ge$ 1, CPT status $\ge$ 1, elevated CEA levels, and reduced HGB levels were identified as significant independent predictors associated with poorer RFS.

**Table 4:** Risk factors of recurrence outcome: univariate and multivariate COX analyses

Variable	Univariate			Multivariate		
	HR	95$\%$ CI	*p*	HR	95$\%$ CI	*p*
Sex, male vs female	0.796	(0.515–1.230)	0.3			
Age, years, $\le$ 50 vs $\ge$ 50	0.741	(0.531–1.030)	0.07			
BMI, $\le$ 18.50	Ref					
BMI, 18.51–23.99	0.98	(0.306–3.130)	0.97			
BMI, 24.00–27.99	0.887	(0.277–2.830)	0.83			
BMI, $\ge$ 28.00	1.09	(0.329–3.60)	0.89			
ASA score 3 vs 2	1.27	(0.906–1.770)	0.16			
ECOG PS score, $\ge$ 1 vs 0	2.08	(1.170–3.680)	0.012	1.89	(1.050–3.40)	0.034
CPT status, $\ge$ 1 vs 0	3.06	(1.780–5.250)	< 0.001	2.36	(1.330–4.190)	0.003
aCCI, $\ge$ 5 vs < 5	1.110	(0.804–1.540)	0.51			
ALBI score, 1	Ref					
ALBI score, 2	1.17	(0.822–1.650)	0.39			
ALBI score, 3	2.63	(0.812–8.510)	0.1			
IWATE criteria difficulty, Low	ref					
IWATE criteria difficulty, Intermediate	1.11	(0.712–1.720)	0.65			
IWATE criteria difficulty, advanced	1.32	(0.795–2.180)	0.28			
IWATE criteria difficulty, expert	3.67	(0.864–15.60)	0.07			
Surgical approach, RLR vs LLR	0.599	(0.412–0.871)	0.007	0.586	(0.393–0.874)	0.008
AFP, ng/mL, > 10 vs $\le$ 10	1.08	(0.781–1.490)	0.64			
CA125, U/mL, $\le$ 12.46 vs > 12.46	0.68	(0.482–0.959)	0.02	0.819	(0.574–1.170)	0.27
CA19-9, U/mL, $\le$ 21.86 vs > 21.86	0.519	(0.342–0.788)	0.002	0.746	(0.479–1.160)	0.19
CEA, ng/mL, $\le$ 3.59 vs > 3.59	0.502	(1.010–1.060)	< 0.001	0.51	(0.347–0.748)	< 0.001
HGB, g/L, $\le$ 142 vs > 142	1.77	(1.260–2.470)	< 0.001	1.7	(1.210–2.40)	0.002
PLT, 10^9^/L $\le$ 149 vs > 149	1.41	(1.020–1.950)	0.03	1.17	(0.836–1.630)	0.36

The long-term results of liver segments I, VII, and VIII and the combined analysis of the three sites were also compared, and the results showed no statistically significant difference in either RFS or OS (outlined in Appendix Table 5, 6, 7, 8).

### Learning curve analysis

**Fig. 2:**
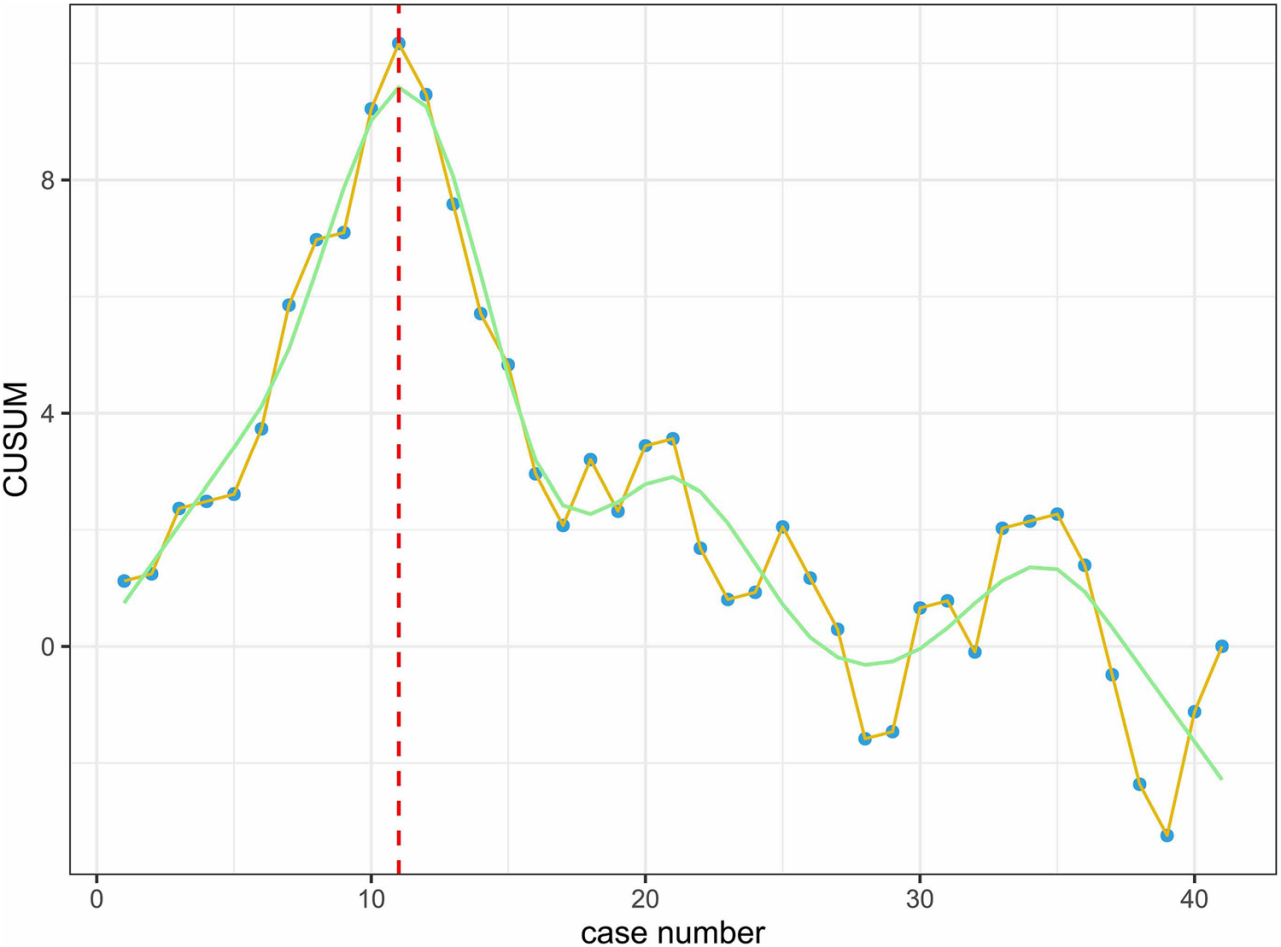
The learning curve of robotic liver resection

At our center, the initial RLR case was conducted in July 2016. To reduce bias, this evaluation used data after PSM analysis. The data were collected from the entire medical center, a total of 4 liver surgery departments, mainly by eight surgeons. All the surgeons who performed the minimally invasive surgery had a master’s degree or above in surgery and had an average of more than 5 years of experience in minimally invasive surgery. They already had extensive experience in laparoscopic and open surgery before robotic surgery was introduced into the medical center. To mitigate biases, data post-PSM analysis were utilized in this assessment. To maintain consistency, 56 patients who underwent RLR performed by a surgeon other than Dr. Zhang, the most frequent practitioner of RLR surgeries during the study period, were excluded from this evaluation. Consequently, a total of 41 patients in the RLR group were considered for this analysis. The CUSUM curve for RLR is depicted in Fig. 2. It is worth noting that the transition from positive to negative slope in the CUSUM curve occurs after the 11th case, indicating that the proficiency period of the learning curve is achieved after approximately 11 RLR procedures.

## Discussion

To our knowledge, this study represents the largest single-center, retrospective, observational cohort study in China at the time of study registration, investigating the long-term prognostic outcomes, short-term outcomes, and perioperative outcomes of consecutive patients with HCC treated with either RLR or LLR. Our study shows that OS was comparable between RLR and LLR, and RFS was improved in the RLR group after a PSM analysis based on clinical, oncologic, and technical criteria. The similar OS between the two groups may be attributed to cancer staging and the effectiveness of treatment after relapse. Most patients in our study had early stage HCC and were still in middle age with good overall physical condition, who inherently have better prognoses and longer OS. This could mask the potential survival benefits of the RLR approach in more advanced stages. In addition, in most situations, both groups received similar, aggressive, and effective treatment when they relapsed, leading to undifferentiated survival outcomes.

The higher RFS rate in the RLR group could be attributed to several key factors. A previous European multicenter comparative study by Lim et al. analyzed data from patients who underwent a multicenter comparative study for HCC [17]. The 3-year RFS rates for 3D-laparoscopic and robotic surgeries were 24% and 48% (*p* = 0.18), respectively. Although there was no statistical difference, the RFS rate in the RLR group was twice that in the LLR group, which is evidence that cannot be ignored. There are also single-center studies showing that the 3-year RFS rate was 50% in the LLR group and 64% in the RLR group (*p* = 0.30), suggesting a 14% higher recurrence-free survival rate in the RLR group compared to the LLR group [18]. Considering that these studies began and concluded approximately 5 years earlier than our own, and given the faster pace of technological iteration for RLR compared to LLR, there has been a substantial increase in the number of surgical procedures each year, as well as an expansion of the indications for robotic liver surgery [19, 20]. Various clinical advantages of RLR have been continuously reported, and its actual clinical efficacy is increasing [19, 20]. This may partly explain why the RLR group exhibits a superior recurrence-free survival rate compared to the LLR group. The rapid development of robotic surgical systems aims to reduce human error and provide feedback during the execution of standardized surgical procedures [21]. Due to the unique features of the robotic surgical system, such as motion scaling and enhanced three-dimensional vision [2, 22], providing superior visualization and high flexibility during surgery, allowing a more thorough exploration of the area around the tumor bed, facilitating finer cuts and suturing, which reduces the likelihood of tiny tumor residuals and thus the risk of recurrence. Our research shows that the rate of positive surgical margins in the RLR group was 1.0 % whereas in the LLR group, it was 4.1$\%$. The RLR group’s rate was approximately one-quarter that of the LLR group, suggesting that RLR may facilitate a higher rate of complete tumor resection (R0) and thereby mitigate tumor recurrence. In addition, the robot platform has a unique tremor filtering function [2, 22], which can reduce the involuntary tremor of the hand, improve the stability of the operation, reduce errors during the operation, and make it easier to cope with various situations during the operation. This fact is further emphasized in our study by showing a conversion rate of 2.1$\%$ in the RLR group and 7.4$\%$ in the LLR group during surgery. Conversion to open surgery often leads to increased trauma, prolonged postoperative recovery, and increased risk of complications, which is more likely to lead to an increased probability of tumor recurrence. Our results of surgical complications showed that the incidence of Ascites was 20.6$\%$ in the RLR group and 25.4$\%$ in the LLR group. The incidence of Pleural effusion was 28.9$\%$ in the RLR group and 34.0$\%$ in the LLR group. Intra-abdominal infection in LLR was more than twice that in RLR (8.6$\%$ vs 4.1$\%$). Complications, such as pleural effusion, ascites, or postoperative infection, may weaken the patient’s immune system, making the patient more susceptible to mutation and invasion of tumor cells. In addition, surgical complications can prevent patients from completing a series of postoperative treatments, such as interventional embolization or radiotherapy, which help to kill residual tumor cells and reduce the risk of recurrence. Compared with LLR, RLR can reduce the incidence of surgical complications and the risk of recurrence to a certain extent. Although RLR only improved RFS compared with LLR, relatively longer RFS means a healthier physical and mental state and a higher quality of life for patients. From a different perspective, RLR is only about two decades old compared to the decades-long record of LLR [23, 24] and has already achieved similar results in various aspects. The enhanced potential of LLR may still exist. However, it is limited, and robot-assisted surgery is entering a phase of rapid development with continuous optimization of the surgical learning curve [16, 25]. The prospects of RLR are undoubtedly excellent, and its expected long-term oncological benefits are destined to exceed those of conventional surgical approaches.

High-quality multicenter studies have indicated that RLR can lead to reduce blood loss, fewer conversions to open surgery, decreased postoperative morbidity, and shortened hospital stays compared with LLR [4, 5, 26–29]. These findings support the argument for RLR as the surgical method of choice, especially in improving surgical safety and facilitating rapid recovery. However, it should be noted that other studies have reached different conclusions, stating that RLR may increase blood loss, increase the need for blood transfusion, increase major postoperative morbidity, and increase mortality within 30 and 90 days compared with LLR [30]. In our analysis of the entire patient cohort, we did not observe significant differences between RLR and LLR groups in the primary perioperative outcomes, such as blood loss, transfusion rate, and length of hospital stay, despite theoretical differences in surgical approach between RLR and LLR. In addition, readmission rates within 30 days, reoperation rates within 30 days, and mortality rates within 90 days were similar between RLR and LLR groups. Considering that the main cause of liver cancer in China is HBV infection, this is quite different from Western countries. In addition, the health status of patients, the experience of surgeons, and the local medical resources are also very different, so we are more inclined to the view that robotic hepatectomy is not inferior to laparoscopic hepatectomy. We believe that both approaches can be safely used in the treatment of hepatocellular carcinoma with good results. This is also consistent with Peng Zhu’s report (3). In addition, our results showed that the operation time was longer in the RLR group than in the LLR group, which is also consistent with the previous studies [31, 32], and we believe that this is intricately linked to the learning curve. This study commenced with the initial RLR case for HCC in our center, intentionally not starting post the proficiency period of the learn. We aimed to genuinely demonstrate the results of a new surgical technique from its introduction to gradual application compared to the established traditional technique. While, as LLR in our center began many years earlier than RLR, the LLR procedures are considerably more established, leading to the actual results being in the surgical proficiency phase, whereas some actual results of RLR do not reach the expected surgical maturity phase. Consequently, we speculate that RLR in the surgical proficiency stage may outperform LLR in multiple aspects in future studies.

To map the learning curve of individual surgeons in this study, we specifically employed multidimensional CUSUM analysis. Our institution holds considerable experience in hepatectomy, having performed numerous procedures, particularly utilizing RLR since the introduction of the da Vinci Surgical System in 2016. This vast experience positions our institution well for this comparative analysis. We utilized operative time, estimated blood loss, postoperative complications, and postoperative hospital length of stay as evaluation indicators of surgical competency. The fitted curve indicated that the proficiency period began after the 11th case, marking the proficiency stage of the learning curve. A prior systematic review reported a decline in the number of cases needed to achieve surgical proficiency from 48.3 in 1995 to 23.8 in 2015 [33]. Additionally, the international consensus guidelines on robotic liver resection in 2023 suggest that an experienced surgeon typically requires around 25 consecutive cases to surpass the learning curve for major RLR and 15 cases for minor RLR, further emphasizing the influence of LLR experience on the RLR learning curve [34]. Due to variations in assessment criteria for evaluating surgeons’ proficiency across different studies and individual differences such as the actual number of surgeries and individual learning capabilities of our doctors, we consider the results of this study acceptable. However, because this learning curve is individual-specific, its general applicability may be limited. Nonetheless, it can serve as a valuable reference, particularly in the epidemiological context of liver cancer, such as in China.

As previously mentioned, RLR demonstrates a long-term oncological advantage compared to laparoscopic liver resection LLR. These findings align with our expectation, because we believe that the robotic surgical system offers superior technical precision in operation and resection, potentially reducing the tendency for tumor recurrence. Moreover, the potential interference induced by economic factors should not be overlooked. As previously mentioned, the economic circumstances of patients in the LLR group might be inferior to those in the RLR group. Consequently, postoperative aspects, such as diet recovery, medication adherence, and environmental support, may be more favorable in the RLR group, potentially contributing to a longer RFS compared to the LLR group.

This study encompasses several limitations. Primarily, its retrospective nature serves as a significant constraint and may be associated with information and selection biases. Although PSM analysis was employed to mitigate selection bias, residual selection bias due to unmeasured or unknown confounders remains inevitable in the absence of randomization. Moreover, as a single-center study, it may not capture potential variations in patient management across various high-volume institutions, as differences in surgical experience and perioperative protocols are minimized.

To address these challenges, future endeavors should focus on conducting multinational, multicenter cohort studies to ensure sample diversity, minimize unknown selection biases, and guarantee external validity. Additionally, single-target value methodologies may also be considered for application in future research.

## Conclusions

The findings of this study confirm the feasibility and safety of RLR as a surgical treatment for HCC. Patients who underwent RLR for HCC exhibited improved RFS, while the OS was found to be comparable to that of LLR. This research highlights the oncological viability of RLR in treating HCC and lays the groundwork for future randomized controlled trials.

## Supplementary Information

Below is the link to the electronic supplementary material.

Supplementary file 1 (docx 36 KB)

## Figures and Tables

**Fig. 3:**
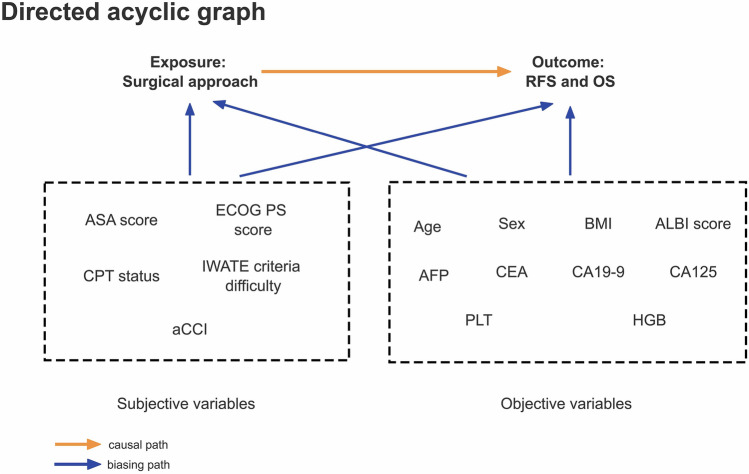
The directed acyclic graphs (DAG) for surgical approach (exposure) and OS and RFS (outcomes). As illustrated, confounding variables are divided into two categories: subjective variables, which are primarily influenced by individual perceptions, and objectively characterized variables

## Data Availability

The datasets generated during and/or analyzed during the current study are not publicly available, but are available from the corresponding author on reasonable request.

## Appendix 1

See Fig. 3 and Tables 5, 6, 7, and 8.

**Table 5:** Perioperative outcome, postoperative short-term outcome, and postoperative long-term outcome of liver segment I

Variable	Before PSM			After PSM		
	RLR (*n* = 7)	LLR (*n* = 14)	*p*	RLR (*n* = 6)	LLR (*n* = 8)	*p*
EBL, median (IQR), mL	200 (20–200)	175.0 (50.0–575)	0.63	125.0 (20–525)	125.0 (62.5–800)	0.74
Operative time, median (IQR), mins	143 (110–216)	183 (119.75–312.5)	0.33	130.0 (101.25–196.75)	146.5 (92.75–378.0)	0.70
Postoperative hospital length of stay, median (IQR), days	7 (5–8)	11 (8–13.25)	0.007	7.5 (5–8)	9.5 (6.5–14.0)	0.09
30-day readmission rate			0.71			0.35
No	5 (71.5)	12 (85.7)		4 (66.6)	8 (100)	
Yes	2 (28.5)	2 (14.3)		2 (33.4)	0	
30-day reoperation rate			0.43			0.078
No	5 (71.5)	11 (78.5)		4 (66.6)	7 (87.5)	
Yes	2 (28.5)	3 (21.5)		2 (33.4)	1 (12.5)	
90-day mortality			0.47			
No	7 (100)	13 (92.8)		6 (100)	8 (100)	
Yes	0	1 (7.2)		0	0	
RFS, 95$\%$CI, month	45.46 (24.21–66.72)	44.18 (31.04–57.31)	0.63	52.38 (32.35–72.40)	50.71 (32.02–69.41)	0.68
OS, 95$\%$CI, month	50.91 (27.75–74.06)	61.27 (48.80–73.73)	0.15	58.72 (37.37–80.08)	54.63 (34.95–74.30)	0.60

**Table 6:** Perioperative outcome, postoperative short-term outcome, and postoperative long-term outcome of liver segment VII

Variable	Before PSM			After PSM		
	RLR (*n* = 5)	LLR (*n* = 54)	*p*	RLR (*n* = 3)	LLR (*n* = 32)	*p*
EBL, median (IQR), mL	400 (50–1400)	100 (50–425)	0.21	400 (50–400)	100 (50–375)	0.27
Operative time, median (IQR), mins	219 (162.5–230.0)	183 (133.75–262.5)	0.54	210.0 (115–210)	183.5 (135.0–293.75)	0.86
Postoperative hospital length of stay, median (IQR), days	8 (6–11)	8 (7–11)	0.86	8 (8–8)	8 (7–10.5)	0.40
30-day readmission rate			0.94			0.43
No	4 (80)	47 (87.0)		2 (66.7)	28 (87.5)	
Yes	1 (20)	7 (13.0)		1 (33.3)	4 (12.5)	
30-day reoperation rate			0.66			0.32
No	4 (80)	44 (81.4)		2 (66.7)	27 (84.3)	
Yes	1 (20)	10 (18.6)		1 (33.3)	5 (15.7)	
90-day mortality						
No	5 (100)	54 (100)		3 (100)	32 (100)	
Yes	0	0		0	0	
RFS, 95$\%$CI, month	44.87 (34.04–55.71)	54.25 (35.66–72.83)	0.52	47.0 (9.76–84.24)	51.98 (37.60–66.36)	0.85
OS, 95$\%$CI, month	66.50 (44.28–86.7)	65.50 (57.70–75.30)	0.74	53.0 (18.35–87.64)	76.23 (68.63–83.83)	0.44

**Table 7:** Perioperative outcome, postoperative short-term outcome, and postoperative long-term outcome of liver segment VIII

Variable	Before PSM			After PSM		
	RLR (*n* = 14)	LLR (*n* = 65)	*p*	RLR (*n* = 12)	LLR (*n* = 43)	*p*
EBL, median (IQR), mL	50 (50–300)	100 (50–200)	0.89	50 (50–275)	100 (50–200)	0.81
Operative time, median (IQR), mins	210.0 (142.7–256.0)	166.0 (113.5–254.5)	0.22	280.5 (209.0–518)	175.0 (125.0–256.0)	0.51
Postoperative hospital length of stay, median (IQR), days	7 (5.8–8)	8 (7–11)	0.06	8 (7–12.1)	10 (8–12.6)	0.11
30-day readmission rate			0.44			0.6
No	13 (92.8)	58 (89.2)		11 (91.6)	38 (88.3)	
Yes	1 (7.2)	7 (13.0)		1 (8.4)	5 (11.7)	
30-day reoperation rate			0.32			0.75
No	13 (92.8)	55 (84.6)		11 (91.6)	37 (86.0)	
Yes	1 (7.2)	10 (15.4)		1(8.4)	6 (14.0)	
90-day mortality			0.42			
No	14 (100)	64 (98.4)		12 (100)	43 (100)	
Yes	0	1 (1.6)		0	0	
RFS, 95$\%$ CI, month	63.05 (50.09–76.02)	45.83 (37.17–54.49)	0.78	62.77 (47.24–78.31)	47.72 (38.25–57.19)	0.28
OS, 95$\%$ CI, month	69.93 (58.27–81.60)	71.27 (66.08–76.47)	0.95	68.02 (54.24–81.80)	73.69 (68.93–78.44)	0.37

**Table 8:** The combined analysis of liver segment I, segment VII, and segment VIII

Variable	Before PSM			After PSM		
	RLR (*n* = 23)	LLR (*n* = *n* = 111)	*p*	RLR (*n* = 20)	LLR (*n* = 66)	*p*
EBL, median (IQR), mL	100 (50–300)	100 (50–300)	0.63	75 (50–300)	100 (50–225)	0.70
Operative time, median (IQR), mins	210.0 (117.0–220.0)	175.0 (127–253)	0.56	216.75 (192.5–216.75)	175.0 (126.5–262.5)	0.92
Postoperative hospital length of stay, median (IQR), days	8 (7–11.8)	11 (8–13)	0.21	7 (6–8)	8 (7–9.3)	0.98
30-day readmission rate			0.88			0.37
No	19 (82.6)	98 (88.2)		16 (80)	60 (91.0)	
Yes	4 (17.4)	13 (11.7)		4 (20)	6 (9.0)	
30-day reoperation rate			0.45			0.18
No	19 (82.6)	98 (88.2)		16 (80)	58 (87.8)	
Yes	4 (17.4)	13 (11.7)		4 (20)	8 (12.1)	
90-day mortality			0.51			
No	23 (100)	109 (98.1)		20 (100)	66 (100)	
Yes	0	2 (1.9)		0	0	
RFS, 95$\%$CI, month	55.15 (44.09–66.20)	46.20 (39.09–53.32)	0.29	56.67 (45.03–68.30)	53.0 (43.34–62.66)	0.77
OS, 95$\%$CI, month	62.20 (50.63–73.76)	71.03 (65.47–76.58)	0.35	68.02 (54.24–81.80)	76.06 (68.93–78.44)	0.11
